# Risk Factors for Early Return Visits to the Emergency Department in Patients Presenting with Nonspecific Abdominal Pain and the Use of Computed Tomography Scan

**DOI:** 10.3390/healthcare9111470

**Published:** 2021-10-30

**Authors:** Fei-Fei Flora Yau, Ying Yang, Chi-Yung Cheng, Chao-Jui Li, Su-Hung Wang, I-Min Chiu

**Affiliations:** 1 Department of Emergency Medicine, Kaohsiung Chang Gung Memorial Hospital, Kaohsiung 833, Taiwan; f_ay_e@hotmail.com (F.-F.F.Y.); kh5318662@gmail.com (Y.Y.); qzsecawsxd@cgmh.org.tw (C.-Y.C.); chaojui@hotmail.com (C.-J.L.); 2 Department of Computer Science and Engineering, National Sun Yat-Sen University, Kaohsiung 804, Taiwan; 3 Division of Hepatogastroenterology, Department of Internal Medicine, Chi-Mei Medical Center, Tainan 710, Taiwan; yourlove1213@gmail.com

**Keywords:** nonspecific abdominal pain, emergency department, return visit, computed tomography scan

## Abstract

Over a quarter of patients presenting with abdominal pain at emergency departments (EDs) are diagnosed with nonspecific abdominal pain (NSAP) at discharge. This study investigated the risk factors associated with return ED visits in Taiwanese patients with NSAP after discharge. We divided patients into two groups: the study group comprising patients with ED revisits after the index ED visit, and the control group comprising patients without revisits. During the study period, 10,341 patients discharged with the impression of NSAP after ED management. A regression analysis found that older age (OR [95%CI]: 1.007 [1.003–1.011], *p* = 0.004), male sex (OR [95%CI]: 1.307 [1.036–1.650], *p* = 0.024), and use of NSAIDs (OR [95%CI]: 1.563 [1.219–2.003], *p* < 0.001) and opioids (OR [95%CI]: 2.213 [1.643–2.930], *p* < 0.001) during the index visit were associated with increased return ED visits. Computed tomography (CT) scans (OR [95%CI]: 0.605 [0.390–0.937], *p* = 0.021) were associated with decreased ED returns, especially for those who were older than 60, who had an underlying disease, or who required pain control during the index ED visit.

## 1. Introduction

Abdominal pain is the most common chief complaint among adult patients visiting emergency departments (EDs). Abdominal pain is the complaint behind 5 to 10% of ED visits annually and continues to pose great diagnostic challenges for emergency physicians due to its widely variable clinical manifestations and the large number of possible differential diagnoses [1,2,3]. Its etiology can range from benign to undifferentiated to life-threatening diseases. Over a quarter of patients presenting with abdominal pain while visiting the ED were ultimately diagnosed with nonspecific abdominal pain (NSAP) at discharge [4,5]. Although a specific diagnosis is frequently difficult to make at the ED, abdominal pain is one of the most common causes of ED revisits [6,7,8]. One study pointed out that 4% of yearly ED return visits are for NSAP [9], and a study of 1411 patients discharged from the hospital with NSAP found that 8% of them were readmitted with abdominal pain within one year [10].

Revisits to the ED are medical costs-consuming, frustrating to both patients and caregivers, and may add strain to the already overburdened healthcare environment [11,12]. Reducing the unscheduled revisit rate requires understanding the risk factors associated with return visits among ED patients. Several studies have sought to identify the risk factors for return ED visits in the general ED population [13,14,15,16,17]. Recently, several revisit prediction models have been developed for children and elderly patients with specific diseases, such as urinary tract infection and asthma [18,19,20,21,22,23]. It is imperative for emergency physicians to identify the risk of revisiting before discharge among patients presenting with NSAP, one of the most common complaints associated with return visits. This study investigates the risk factors associated with return ED visits in patients with NSAP after discharge, which (to the best of our knowledge) no study has yet investigated. We also analyze the use of computed tomography (CT) scans in these patients.

## 2. Method

### 2.1. Study Setting and Patient Population

We conducted a retrospective database study at two tertiary medical centers in Taiwan from 1 January 2016 to 31 December 2018. One hospital was located in northern Taiwan, and the other was located in southern Taiwan. They were both the largest medical centers in their metropolitan areas, with 80,000 and 110,000 average ED visits annually, respectively. The study protocol was approved by the institutional review board of both hospitals (IRB number 202002043B0; approved on 1 December 2020).

**Figure 1:**
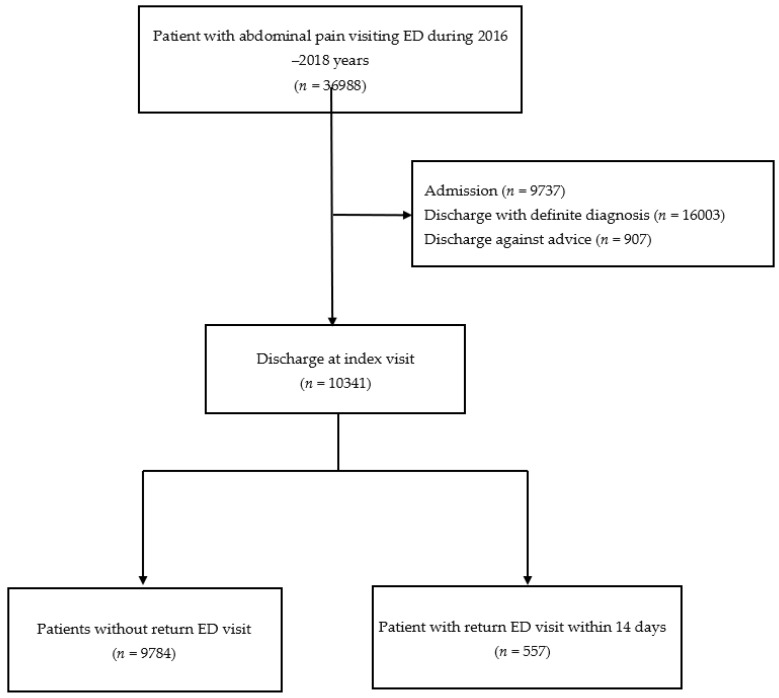
Patients’ inclusion flow chart.

The sample comprised patients who had presented to the ED with abdominal pain and were discharged directly from the ED. We identified NSAP using the International Classification of Disease Code, 10th version (ICD-10) code R10. Patients were excluded if they were under 18 years old, had a prior ED visit within the 14 days preceding the index ED visit, had a history of trauma, had a definite diagnosis related to abdominal pain, transferred to another hospital, or discharged against medical advice. The patient selection flowchart is presented in Figure 1. Patient and physician records and information were collected from research databases at the study hospitals and were anonymized and de-identified before the analysis.

### 2.2. Outcome Measurement

We investigated the characteristics and risk factors of return ED visits among patients presenting with NSAP by dividing the patients into two comparison groups: the study group, composed of patients with return ED visits after the index ED visit; and the control group, composed of patients without revisits. The index ED visit was defined as the first ED visit for a unique patient with no prior visit during the preceding 14 days.

A recent study that determined the time of an ED revisit suggested that nine days was the most reasonable cutoff for the identification of acute ED revisits [24]. To include the largest possible number of return visits, we defined a return visit as an ED revisit within 14 days after discharge from the index ED visit. Only the first revisit was considered for patients with multiple ED revisits within 14 days.

### 2.3. Statistical Analysis

Data on patient demographics, underlying medical diseases, vital signs at ED admission and discharge, and length of ED stay were all collected for risk factor analysis. Data regarding ED examinations, such as laboratory tests, X-rays, and computed tomography (CT) scans were also included as parameters. CT scans with or without contrast medium administration were both included for analysis. We also analyzed the use of pain control in the ED because it may reflect the severity of the patient’s condition during ED observation.

Data were presented as medians (interquartile range [IQR]) for continuous variables and as proportions for nominal variables. We performed a Mann–Whitney U test and two-tailed chi-square analysis to determine the risk factors that correlated with return ED visits in patients with abdominal pain. We developed multivariable regression models to evaluate the odds ratio of risk factors for ED revisit in the study groups, controlling for age, sex, comorbidities, and other associated confounding factors. Statistical significance was set at *p* less than 0.05. Statistical analyses were performed using SPSS Statistics for Mac, version 26, IBM^®^, U.S.

## 3. Results

**Figure 2:**
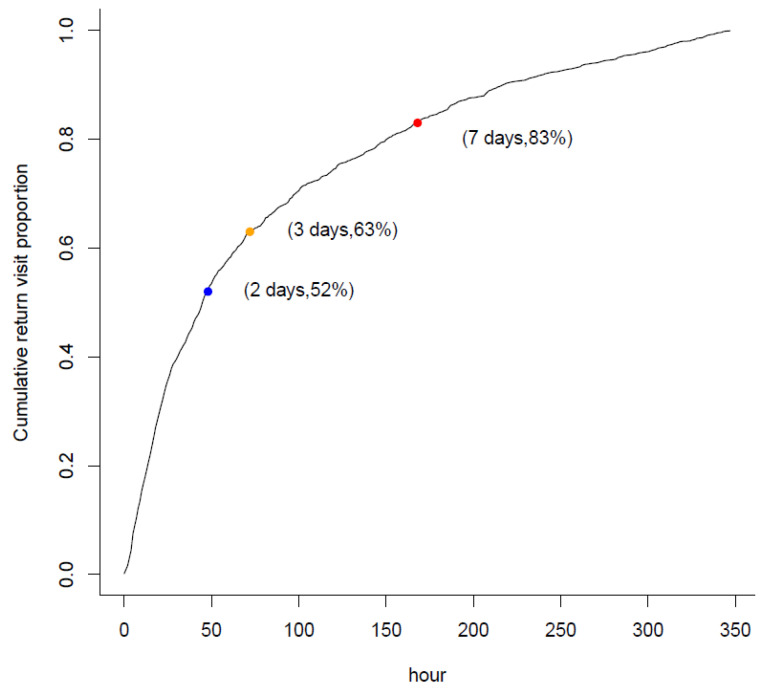
Cumulative proportion of return visit by hours.

During the study period, 561,630 patients visited an ED. Among them, 36,988 (6.6%) presented with abdominal pain, and 10,341 were discharged with the impression of NSAP after index ED management. Of the discharged patients, 557 patients who returned to the ED within 14 days comprised the study group, and the other 9784 patients formed the comparison group (see Figure 1). A total of 63% (*n* = 351) of the patients returned to the ED within three days after the index ED discharge, and 83% (*n* = 462) returned within seven days, representing most revisit patients (see Figure 2).

Table 1 presents the demographics and characteristics of the patients with and without return visits. We found that patients with return ED visits were older (50 (36–65) vs. 42 (31–58); *p* < 0.001) and predominantly male (47.0% vs. 37.6%; *p* < 0.001). Median arterial pressure (MAP) at admission (104 (92–117) vs. 100 (89–113); *p* < 0.001) and discharge (95 (85–106) vs. 92 (82–103); *p* < 0.001) were higher in the return visit group. Higher temperature at discharge (36.5 (36.1–36.8) vs. 36.3 (36.0–36.8) °C; *p* = 0.029) was noted in patients with return visits. No significant difference in length of stay during the index ED visit was observed (1.7 (1.2–2.7) vs. 1.8 (1.2–3.0) h; *p* = 0.190).

A higher proportion of chronic underlying diseases, including Hypertension (24.8% vs. 18.8%, *p* < 0.001), Diabetes Mellitus (DM; 11.0% vs. 6.9%, *p* < 0.001), Chronic Kidney Disease (CKD; 3.8% vs. 2.1%; *p* = 0.015), and Malignancy (10.4% vs. 6.2%; *p* < 0.001), was noted in patients with return ED visits.

Of all included patients, 1339 of them received CT scans during their index ED visit, in which 74.8% were without contrast medium administration. We found no statistical differences in the proportion of CT, X-ray, and laboratory tests performed during ED examination between the two groups. More patients in the study group received pain control with non-steroidal anti-inflammatory drugs (NSAIDs; 55.3% vs. 48.9%; *p* < 0.001) or opioids (22.4% vs. 12.2%; *p* < 0.001) during their index ED visit.

After adjusting for confounding factors in the multivariate regression analysis, we found that older age (OR [95%CI]: 1.007 [1.003–1.011], *p* = 0.004), male sex (OR (95%CI): 1.307 [1.036–1.650]; *p* = 0.024), and use of NSAIDs (OR [95%CI]: 1.563 [1.219–2.003]; *p* < 0.001) or opioids (OR [95%CI]: 2.213 [1.643–2.930]; *p* < 0.001) during the index visit were associated with an increased rate of ED return visits within 14 days. In contrast, CT scanning (OR [95%CI]: 0.605 [0.390–0.937]; *p* = 0.021) was associated with a decreased rate of return ED visits (see Table 2). Other ED examinations, such as X-rays (OR [95%CI]: 0.819 [0.642–1.04]; *p* = 0.106) and laboratory tests (OR [95%CI]: 0.992 [0.763–1.290]; *p* = 0.954), were not associated with return ED visits after regression analysis.

We performed a subgroup analysis to examine the utility of CT scans in reducing the rate of ED revisits in various patient populations (see Table 3). CT scanning was associated with decreased return visits in patients aged over 60 years old (OR [95%CI]: 0.554 [0.302–0.978]; *p* = 0.036) but not in patients aged under 60 (OR [95%CI]: 0.789 [0.547–1.137]; *p* = 0.204). The odds ratio was even lower in patients aged over 80 (OR [95%CI]: 0.477 [0.132–1.730]; *p* = 0.260), while no statistical difference was observed. Regarding gender differences, CT scanning was not associated with ED revisits in either males (OR [95%CI]: 0.765 [0.496–1.180]; *p* = 0.226) or females (OR [95%CI]: 0.656 [0.419–1.026]; *p* = 0.065). Finally, CT scanning was associated with decreased return visits in patients who had underlying diseases (OR [95%CI]: 0.423 [0.221–0.812]; *p* = 0.01) or who had received pain control during the ED visit (OR [95%CI]: 0.609 [0.419–0.884]; *p* = 0.009).

Table 4 demonstrates the outcomes of return visits in NSAP patients with or without a CT scan study in their index ED visit. Of all return visits, 69.7% patients discharged, while 14.5% of them underwent surgery after revisit. The most common indications for operation were acute cholecystitis (27.1%), acute appendicitis (20.3%), and bowel obstruction (18.6%). Receiving a CT scan study during their index ED visit was associated with less admission (16.4% vs. 31.9%, *p* = 0.002) and operation (8.2% vs. 15.3%, *p* = 0.008). More patients who receive a CT scan during their index ED visit were diagnosed with NSAP again in their return visit compared to those who did not receive a CT scan during their index ED visit (74.5% vs. 51.5%, *p* < 0.001).

## 4. Discussion

This study sought to identify the risk factors for return ED visits within 14 days after discharge in patients diagnosed with NSAP. Studies have demonstrated that 25 to 46% of patients admitted to the ED with abdominal pain are discharged with a diagnosis of NSAP [1,3,4,5,25,26,27,28]. Approximately 28% of the patients examined in this study were discharged with NSAP. Among these, 5.4% returned to the ED within 14 days.

After comparing patients with and without ED return visits within 14 days of the index visit, we found several risk factors that could be readily identified by the emergency physician during the initial visit. ED return visits were significantly more likely in patients with the following: older age, male sex, higher MAP at admission and discharge, higher body temperature at discharge, history of chronic disease (e.g., Hypertension, DM, CKD, Malignancy), and use of NSAIDs or opioids during the index visit.

We discovered that advanced age was a predictor of ED return visits in patients discharged from the ED with NSAP. This finding aligns with previously reported associations between older age and a higher rate of 72-h unscheduled return ED visits [15,29]. Elderly patients have also shown higher rates of admission to unscheduled return ED visits [13,15,29,30]. The factors responsible for repeat ED visits among the elderly were associated with the number of functional and mental problems, such as impaired mobility, incontinence, confusion, and depression [22]. These problems could result in vague nonspecific abdominal complaints, even in patients requiring acute medical or surgical interventions. Cognitive impairment in older patients may also lead to noncompliance with prescriptions or post-discharge instructions.

Revisits were also more common in patients with a higher MAP at admission and discharge and higher body temperature at discharge. Revisits were also significantly more common in patients with chronic diseases, such as Hypertension, DM, CKD, and Malignancy. Studies have also reported that DM, CKD, and Malignancy are associated with short-term bounce-back admissions after ED discharge [13,29]. Emergency physicians should pay particular attention to concomitant chronic diseases, as their conditions may seem stable initially but may deteriorate rapidly. Liver cirrhosis was not found to be associated with return ED visits. This result may seem counterintuitive, as patients with liver cirrhosis are expected to be more likely to have serious complications, including ascites, spontaneous bacterial peritonitis, portal hypertension, and variceal bleeding. This result may have occurred because emergency physicians assess these patients more cautiously, so the threshold for discharge may have been higher, and most of these patients were admitted during the index ED visit.

Our data indicate that patients who need the administration of NSAIDs or opioids on initial presentation are a high-risk population for return ED visits, perhaps due to continued abdominal pain and inadequate pain management. Lukens et al. found that 28.4% of patients discharged with undifferentiated abdominal pain described their pain as the same while 3.7% claimed that it became worse within two to three days [4]. Other studies have reported that inadequate pain management remained a problem in the ED [31,32]. Hu et al. also found that a high proportion of patients with unscheduled return ED visits were due to inadequate pain management [13]. Therefore, emergency physicians must achieve sufficient pain management in patients with NSAP before discharge.

The most distinctive finding of the logistic regression analysis was the negative association between CT performance and ED revisits. A similar finding in Patterson et al. revealed that abdominal CT imaging was associated with a lower 30-day revisit rate in patients presenting to the ED with abdominal pain [30].

We performed a subgroup analysis to investigate the effect of CT scans during index visits on return ED visits. Abdominal CT scanning was found to be associated with decreased ED revisits in several subgroups, such as the elderly (age greater than 60 years), those with underlying diseases, and those receiving pain control. This makes sense since these subgroups of patients tend to have more severe illness, and often do not present with the same characteristic symptoms and signs as younger, healthier patients. For example, the classic signs of peritoneal inflammation that are commonly used to distinguish severe intra-abdominal condition may be attenuated in these patients. In previous studies, CT scan were positive in 55–57% of elderly patients presenting to the EDs with abdominal pain [33,34,35,36]. The two most common diagnostic findings were small bowel obstruction and diverticulitis, which CT scan has a high sensitivity to detect.

By contrast, the use of CT scans in patients age ≤ 60 years old was not statistically associated with a return ED visit. Furthermore, CT scan should then be used under caution, especially for female patients in childbearing age, which are vulnerable to excessive radiation exposure [37].

Although our study was not designed to provide a causal explanation for ED return visits, the association between abdominal CT scans and revisits may be an effect of the reassurance a that negative CT result provides, whereby patients feel that potentially life-threatening illnesses have been ruled out. Future studies could investigate the reasons for patients’ decision to return and quantify the effects of negative CT scans on subsequent patient behavior. Even though the findings in this study still stand for CT scans in NSAP patients of older age and underlying disease.

Additionally, the association of CT study and reduced return visit may be a result of selection bias. This hypothesis may be supported by the result from Table 4, that more patients were diagnosed with NSAP and discharge after return visit in those who received a CT scan study during the index ED visit. Patients without CT scan during index visit were associated with higher operation rate in the ED revisit. It is reasonable that most common surgical indications were acute cholecystitis, acute appendicitis, and bowel obstruction, which were all sensitive to be diagnosed with CT scan [38,39]. CT is widely available, quickly performed, and helpful for evaluating multiple intra-abdominal diseases. These advantages give rise to an emerging ED practice of obtaining CT imaging in patients presenting with NSAP. Research has also shown that CT is the rational choice for initial radiological imaging during the ED evaluation of NSAP and should be seriously considered before ED discharge [26]. On the other hand, with the huge progress in utility of point-of-care ultrasound in ED, the combination of ultrasound assessment and other diagnostic tools may replace CT scanning in certain condition.

Our findings have multiple implications for improving the quality of care delivery. As the rate of unscheduled ED revisits is widely used to assess the quality of emergency care, accurately identifying patients at high risk of ED revisits is critical for guiding quality improvement efforts. Our study identified predictors that could be readily evaluated during the initial visit. This should enable emergency physicians to be better able to make informed decisions regarding the disposition and depth of evaluation for those at high risk of a return ED visit. Patients with identified risk factors should be instructed to follow up with their primary care provider to lower the rate of unscheduled ED return visits and improve healthcare resource use. Emergency physicians might also consider lowering the CT threshold in certain patient groups and ensure that sufficient pain management has been achieved before discharge.

Our study has several important limitations. First, as this was a retrospective database study, confounding factors that went unmeasured may have influenced the cause of ED revisits. In our data, medical records reporting on the initial intensity of the abdominal pain, such as the Visual Analogue Score, were incomplete. The decision to order a CT scan, laboratory test, or analgesic therapy may be a proxy for severity of illness and thus, the likelihood to return. Second, the decision to discharge patients can be multifactorial. Patients’ medication adherence, self-care functionality, access to medical services, and social support may change clinicians’ considerations around patient disposition. Third, the patient-based reasons for return visits, such as habitual use of ED and noncompliance with follow-up care, may not be captured in a retrospective database review. Taiwanese are highly likely to bypass outpatient clinics and use the ED for primary care due to the low cost of hospital visits made possible by Taiwan’s national health insurance system. Fourth, we tracked the ED return visits of patients who visited the same hospital. We were unable to identify patients who had already visited an ED at a different hospital before they came to our hospital or who visited a different hospital after they had been seen in our ED. This may have resulted in an underestimation of the return visit rate.

## 5. Conclusions

This retrospective study conducted at two medical centers in Taiwan revealed that older age, male sex, and use of analgesics during index ED visits were significant predictors of return ED visits within 14 days for patients discharged with NSAP. The performance of abdominal CT imaging was associated with decreased ED revisits; these associations were particularly prominent in older patients, those with underlying disease, and those who required pain control during the index ED visit.

## Figures and Tables

**Table 1:** Demographic and clinical characteristics of patient presenting nonspecific abdominal pain with and without return visit.

	with Return Visit Median (IQR)/N (%)	without Return Visit Median (IQR)/N (%)	*p*-Value
Total number of patients	557	9784	
Age, year	50 (36–65)	42 (31–58)	<0.001
Male	262 (47.0)	3680 (37.6%)	<0.001
Vital signs at admission			
Temperature, °C	36.5 (36.1–36.8)	36.4 (36.1–36.6)	0.740
Heart rate, beats per minute	80 (70–92)	81 (72–92)	0.110
MAP, mmHg	104 (92–117)	100 (89–113)	<0.001
Vital signs at discharge			
Temperature, °C	36.5 (36.1–36.8)	36.3 (36.0–36.8)	0.029
Heart rate, beats per minute	75 (67–85)	75 (67–84)	0.950
MAP, mmHg	95 (85–106)	92 (82–103)	<0.001
ED length of stay, hour	1.7 (1.2–2.7)	1.8 (1.2–3.0)	0.190
Chronic disease			
Hypertension	138 (24.8%)	1739 (18.8%)	<0.001
DM	61 (11.0%)	674 (6.9%)	<0.001
CKD	21 (3.8%)	204 (2.1%)	0.015
Liver cirrhosis	9 (1.6%)	123 (1.3%)	0.435
Malignancy	58 (10.4%)	642 (6.2%)	<0.001
ED examination			
Computed tomography	61 (11.0%)	1278 (13.1%)	0.156
X-ray	325 (58.3%)	5822 (59.5%)	0.595
Laboratory test	399 (71.6%)	6678 (68.3%)	0.102
ED medication			
NSAID	308 (55.3%)	4782 (48.9%)	0.003
Opioid	125 (22.4%)	1193 (12.2%)	<0.001

**Table 2:** Logistic regression analysis of clinical characteristic associated with return visit.

Variable	Odds Ratio (95%CI)	*p*-Value
Age	1.007 (1.003–1.011)	0.004
Male	1.307 (1.036–1.650)	0.024
Vital signs		
MAP at admission	1.005 (0.997–1.012)	0.213
MAP at discharge	1.003 (0.993–1.002)	0.559
Fever at discharge (Temperature ≥ 38 °C)	2.129 (0.749–6.056)	0.156
ED medications		
NSAID	1.563 (1.219–2.003)	<0.001
Opioid	2.213 (1.643–2.930)	<0.001
ED examinations		
Computed tomography	0.605 (0.390–0.937)	0.021
X-ray	0.819 (0.642–1.04)	0.106
Laboratory test	0.992 (0.763–1.290)	0.954
Chronic disease		
Hypertension	1.313 (0.905–1.791)	0.102
DM	0.940 (0.633–1.376)	0.759
CKD	1.174 (0.919–1.500)	0.198
Liver cirrhosis	0.992 (0.444–2.217)	0.984
Malignancy	0.946 (0.628–1.426)	0.791

**Table 3:** Subgroup analysis of hazard ratio of CT scan from index visit to return ED visit.

Subgroup	Number	Odds Ratio (95% CI)		*p*-Value
Age ≤ 60 years old	7971	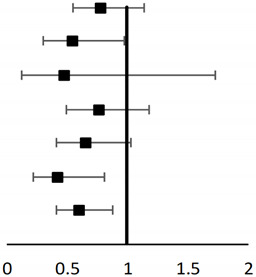	0.789 (0.547–1.137)	0.204
Age > 60 years old	2370		0.544 (0.302–0.978)	0.036
Age > 80 years old	470		0.477 (0.132–1.730)	0.26
Male	3942		0.765 (0.496–1.180)	0.226
Female	6399		0.656 (0.419–1.026)	0.065
Underlying disease	2027		0.423 (0.221–0.812)	0.01
Receive pain control	6098		0.609 (0.419–0.884)	0.009

**Table 4:** Chi-square analysis of clinical outcomes after return ED visit in NSAP patients with or without a CT scan in their index visit.

Outcomes in Return Visit	Without CT Scan during Index ED Visit *N* = 496	With CT Scan during Index ED Visit *N* = 61	*p*-Value
Diagnosed with NSAP	257 (51.8%)	38 (74.5%)	<0.001
Discharge	337 (57.7%)	51 (83.6%)	<0.001
Admission	158 (31.9%)	10 (16.4%)	0.002
Surgery	76 (15.3%)	5 (8.2%)	0.008

## Data Availability

Data were obtained from Chang Gung Research Database and are available from corresponding author with the permission of Chang Gung Medical Foundation.
