# Correction: Yau et al. Risk Factors for Early Return Visits to the Emergency Department in Patients Presenting with Nonspecific Abdominal Pain and the Use of Computed Tomography Scan. *Healthcare* 2021, *9*, 1470

**DOI:** 10.3390/healthcare10010136

**Published:** 2022-01-11

**Authors:** Fei-Fei Flora Yau, Ying Yang, Chi-Yung Cheng, Chao-Jui Li, Su-Hung Wang, I-Min Chiu

**Affiliations:** 1 Department of Emergency Medicine, Kaohsiung Chang Gung Memorial Hospital, Kaohsiung 833, Taiwan; f_ay_e@hotmail.com (F.-F.F.Y.); kh5318662@gmail.com (Y.Y.); qzsecawsxd@cgmh.org.tw (C.-Y.C.); chaojui@hotmail.com (C.-J.L.); 2 Department of Computer Science and Engineering, National Sun Yat-Sen University, Kaohsiung 804, Taiwan; 3 Division of Hepatogastroenterology, Department of Internal Medicine, Chi-Mei Medical Center, Tainan 710, Taiwan; yourlove1213@gmail.com

The authors would like to make corrections to their published paper [1]. The changes are as follows:

1. In Methods, Section 2.1. Study Setting and Patient Population, change the date from “The study protocol was approved by the institutional review board of both hospitals (IRB number 202002043B0; approved on 12 January 2020)” to “The study protocol was approved by the institutional review board of both hospitals (IRB number 202002043B0; approved on 1 December 2020)”.

2. In the back matter, change the date in the **Institutional Review Board Statement** to “approved on 1 December 2020”.

The authors apologize for any inconvenience caused and state that the scientific conclusions are unaffected. The original article has been updated.
